# Selective serotonin reuptake inhibitors and venlafaxine in pregnancy: Changes in drug disposition

**DOI:** 10.1371/journal.pone.0181082

**Published:** 2017-07-14

**Authors:** Andreas Austgulen Westin, Malin Brekke, Espen Molden, Eirik Skogvoll, Olav Spigset

**Affiliations:** 1 Department of Clinical Pharmacology, St Olav University Hospital, Trondheim, Norway; 2 Center for Psychopharmacology, Diakonhjemmet Hospital, Oslo, Norway; 3 Department of Pharmaceutical Biosciences, School of Pharmacy, University of Oslo, Oslo, Norway; 4 Department of Anaesthesiology and Intensive Care, St. Olav University Hospital, Trondheim, Norway; 5 Department of Circulation and Medical Imaging, Norwegian University of Science and Technology, Trondheim, Norway; 6 Department of Laboratory Medicine, Children’s and Women’s Health, Norwegian University of Science and Technology, Trondheim, Norway; Radboud University Medical Centre, NETHERLANDS

## Abstract

**Background:**

Pregnancy may cause changes in drug disposition. The clinical consequences may be profound and even counterintuitive; in some cases pregnant women may need more than twice their usual drug dose in order to maintain therapeutic drug levels. For antidepressants, evidence on drug disposition in pregnancy is scarce. The aim of this study was to determine the effects of pregnancy on serum levels of selective serotonin reuptake inhibitors (SSRIs) and venlafaxine in a large and naturalistic patient material, in order to provide tentative dose recommendations for pregnant women.

**Methods:**

Using patient data from two routine therapeutic drug monitoring (TDM) services in Norway with linkage to the national birth registry, dose-adjusted serum drug concentrations of SSRIs and venlafaxine during pregnancy were compared to the women’s own baseline (non-pregnant) values, using a linear mixed model.

**Findings:**

Overall, the TDM databases contained 196,726 serum concentration measurements from 54,393 women. After data linkage and drug selection (SSRIs or venlafaxine only), we identified 367 analyses obtained from a total of 290 pregnancies in 281 women, and 420 baseline observations from the same women. Serum concentrations in the third trimester were significantly lower than baseline for paroxetine (–51%; 95% confidence interval [CI], –66%, –30%; p<0.001), fluvoxamine (–56%; CI, –75%, –23%; p = 0.004) and citalopram (–24%; CI, –38%, –7%; p = 0,007), and higher than baseline for sertraline (+68%; CI, +37%, +106%; p<0.001). For escitalopram, fluoxetine and venlafaxine concentrations did not change significantly.

**Conclusions:**

For paroxetine and fluvoxamine the pronounced decline in maternal drug serum concentrations in pregnancy may necessitate a dose increase of about 100% during the third trimester in order to maintain stable concentrations. For fluoxetine, venlafaxine, citalopram, escitalopram and sertraline, the present study indicates that dose adjustments are generally not necessary during pregnancy.

## Introduction

Depression in pregnancy is a serious and often overlooked condition. It is estimated to impact 14–23% of pregnant women, which makes it more prevalent in pregnancy than conditions like gestational diabetes (18%) and preeclampsia (3–5%) [1]. Maternal depression may cause a vast range of consequences for the mother and fetus, such as substance abuse, preterm delivery, neonatal intensive care unit admissions, poor bonding between mother and baby, adverse effects on the growth and neurodevelopment of the offspring, and even increased risk of maternal suicide [1, 2]. Therefore, in cases of severe or relapsing depression, the use antidepressants is considered favorable compared to exposing mother and child to untreated depressive illness [1–3].

Choosing the appropriate drug dose for a pregnant woman is a difficult balancing act between optimum maternal treatment and minimal fetal exposure, and is further complicated by the physiological changes that occur during pregnancy. Alterations in maternal body weight, plasma volume, hepatic metabolic capacity and renal function may cause changes in drug disposition [4–7]; thus the right drug dose for a woman prior to conception or for the patient group in general is not necessarily the right dose during pregnancy. For antidepressants, evidence on changes in drug disposition in pregnancy is rather scarce and generally consists of a few studies with 10–20 patients or less for each drug [7–25]. The aim of this study was to elucidate to which extent pregnancy affects serum concentrations of selective serotonin reuptake inhibitors (SSRIs) and venlafaxine in a large target population in a naturalistic setting, in order to provide tentative dose recommendations for pregnant women.

## Methods

### Serum concentration data

After obtaining approval from the Regional Committee for Medical and Health Research Ethics in Mid Norway, the Norwegian Centre for Research Data (Data Protection Official), the Norwegian Directorate of Health and the Medical Birth Registry of Norway (MBRN) publication council, serum concentration data for antidepressants were collected from the two largest TDM services for psychotropic drugs in Norway (i.e. Department of Clinical Pharmacology at St. Olav University Hospital in Trondheim, and Center for Psychopharmacology at Diakonhjemmet Hospital in Oslo). As the Norwegian health care system has a tradition for routine therapeutic drug monitoring (TDM) of psychotropic drugs [26], a considerable amount of TDM data could be retrieved from these databases. The antidepressant TDM data contain serum concentration measurements taken in a naturalistic setting from psychiatry inpatients and outpatients. In addition to measured serum concentrations, the databases contain information obtained from the requisition forms, such as the prescribed antidepressant dose, time of last drug intake, time of blood sampling, and types and doses of concomitant drugs.

### The Medical Birth Registry of Norway (MBRN)

The Medical Birth Registry of Norway (MBRN) is a population based registry containing information on all births in Norway since 1967 [27]. The registry is based on compulsory notification of every birth or late abortion from 12 completed weeks of gestation onwards. The report form includes date of delivery and length of pregnancy as well as other information regarding the mother and infant.

### Data linkage and identification of cases

**Fig 1:**
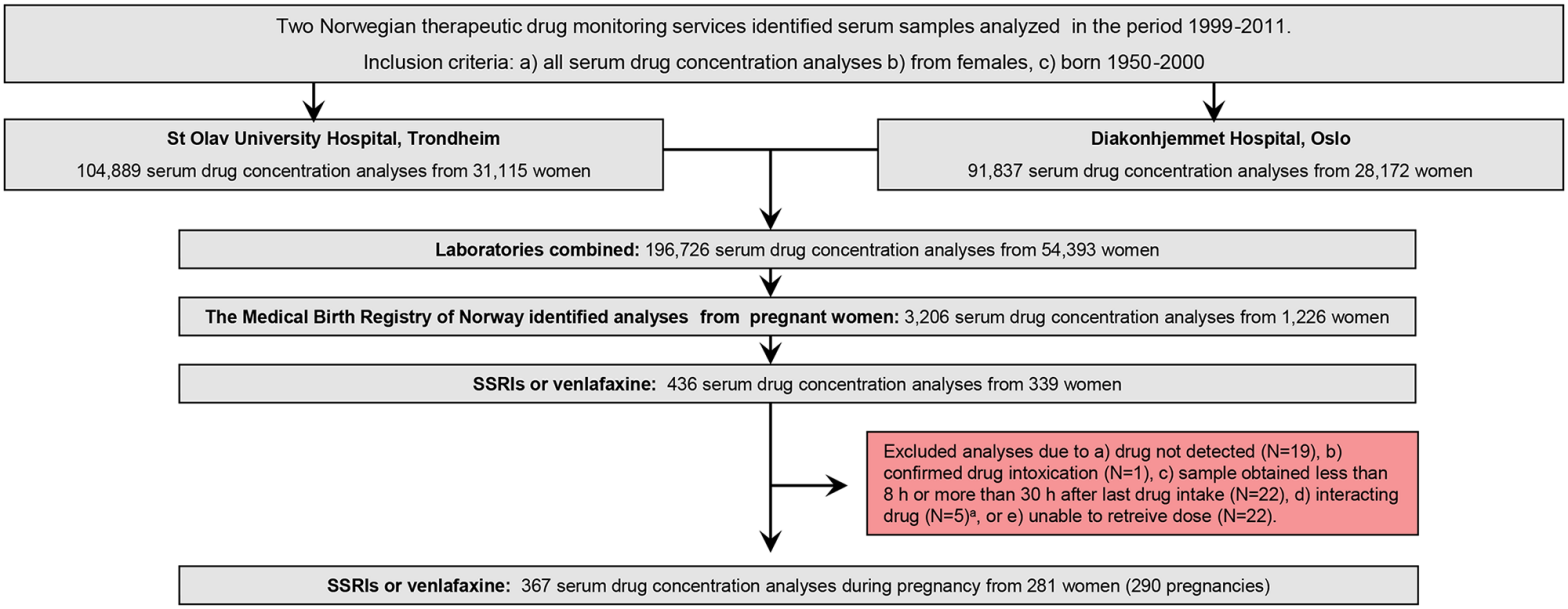
Inclusion flow chart. Sample identification and inclusion of therapeutic drug monitoring samples of selective serotonin reuptake inhibitors and venlafaxine obtained during pregnancy.

First, a combined laboratory TDM file was created, containing all serum concentration measurements (for any drug) in the period October 1999 –December 2011 for all women of reproductive age (i.e. born 1950–2000). The file consisted of a total of 196,726 analyses from 54,393 women (Fig 1). Using the unique 11-digit identification number assigned to all individuals living in Norway, the MBRN could identify all pregnant women in the TDM data set. By applying this procedure, 3206 analyses from 1,226 pregnant women were identified (Fig 1). For the current study we retrieved the following information: the personal identification number, the drug analysed, the measured drug serum concentration, time of last dose, time of sampling, drug dose, concomitant drug use, other clinical information, name of the responsible physician, gestational week at the time of sampling (calculated from the sampling date and the pregnancy onset date as determined by obstetric ultrasound if available, or by last menstruation), and date of delivery.

### Inclusion criteria

The basis of the present study is all samples analyzed for an SSRI (defined as a drug classified in the World Health Organization Anatomical Therapeutic Chemical group N05AB [28]), plus venlafaxine. Then, 436 analyses from 339 pregnant women were available (Fig 1). Analyses were excluded if a) no drug was detected, b) the sample was obtained as a result of drug intoxication, c) the sample was obtained less than 8 hours or more than 30 hours after last drug intake, or d) there was concomitant use of a known interacting drug (i.e. a drug listed in a national drug interaction database as having a major or moderate effect on the plasma concentration on the antidepressant in question [29]). If the requisition form lacked information on drug dose the authors contacted the responsible physician, who attempted to obtain this information from the medical record. If we were unable to retrieve this information, the analysis was excluded. The final data set consisted of 367 serum drug concentrations from 281 women (290 pregnancies) (Fig 1). The individual drugs available are listed in Table 1.

**Table 1:** The study population.

	Number of serum drug concentration analyses			Number of pregnancies	Number of women
	During pregnancy	First twelve weeks following delivery	At baseline		
Escitalopram	110	3	161	97	95
Citalopram	78	3	80	58	58
Fluoxetine	53	2	49	43^a^	41
Sertraline	56	5	52	37	34
Venlafaxine	36	1	44	33^a^	33
Paroxetine	29	6	31	20	19
Fluvoxamine	5	2	3	3	3
Total	367	22	420	290^a^	281^a^^,^^b^^,^^c^

^a^ In one pregnancy both fluoxetine and venlafaxine were analyzed (at different times) due to change in medication.

^b^ One woman used paroxetine in one pregnancy and fluoxetine in another.

^c^ Nine women were pregnant twice.

### Identification of observations from non-pregnant state in the same subjects

Having identified the pregnant women and their individual pregnancy periods in the extracted data file, we used the original TDM databases to retrieve serum concentration measurements before and after pregnancy from the same women, to serve as baseline observations for each of the included subjects. Identical inclusion and exclusion criteria as presented above were applied, and 442 analyses were identified (Table 1). Twenty-two of these were from the first twelve weeks following delivery (i.e. in the “returning to baseline” phase) [19, 22]. These analyses were not used in the statistical model, only for visual comparison. The remaining 420 analyses were used for the statistical comparisons.

### Reference population

In order to provide an estimate of expected antidepressant concentrations in a female reference population, we extracted antidepressant serum concentration data from the same time period for all women aged 18–45 from the St. Olav University Hospital TDM database, using identical inclusion and exclusion criteria as presented above. These data were not included in the statistical analyses, but the 10, 25, 50, 75 and 90 percentile values derived from these data are used for visual comparison purposes. The numbers of analyses upon which these calculations were based were 3265 for escitalopram, 1975 for citalopram, 410 fluoxetine, 1552 for sertraline, 1453 for venlafaxine, 557 for paroxetine and 59 for fluvoxamine.

### Determination of antidepressant concentrations in serum

Quantification of the drug concentrations was performed with liquid chromatography-mass spectrometry/tandem mass spectrometry (LC-MS/LC-MS/MS). The analytical methods have been described in more detail previously [30, 31]. In brief, the drugs were extracted from serum by liquid-liquid extraction, using a mixture of hexane, acetonitrile and/or butanol, or dichloromethane and isopropanol. Thereafter, the analytes were separated on C18 columns using methanol, acetonitrile, formic acid or ammonium acetate as mobile phases, and quantified on LC-MS or LC-MS-MS systems. Calibration curves were constructed for each assay with drug-free human serum by the addition of varying concentrations of the antidepressants and their respective metabolites. All methods were linear in the therapeutic range of the various drugs, and the limits of quantification were generally well below the lower limits of the reference intervals. The inter-day coefficients of variability were in most cases below 10%. During the timespan of the study, some assays had been improved and adjusted, but all modifications were cross-validated with the previous method used for the same drug.

### Data analysis

Serum concentrations in ng/mL were divided by the daily dose used by the woman at the time of sampling, providing a serum concentration/dose ratio, and then multiplied by the defined daily dose (DDD), which is the assumed average maintenance dose per day for that drug used for its main indication in adults [28]. This procedure provides an intra- and interindividually comparable concentration for each drug. All concentrations presented and discussed in this article, including tables and figures, are dose-adjusted to the DDD of the drug. The DDDs for the various drugs are given in Table 2.

**Table 2:** Serum antidepressant concentrations across pregnancy.

	Dose^a^	Estimated serum concentrations												CF^b^
		Base-line	1st trimester		2nd trimester		3rd trimester							
Measure	mg/ day	conc	conc	change	conc	change	conc	CI low	CI high	change	CI low	CI high	p^c^	
		ng/mL	ng/mL	%	ng/mL	%	ng/mL	ng/mL	ng/mL	%	%	%		
Escitalopram	10	9.3	9.4	+1	9.7	+4	9.9	8.0	12.3	+7	-14	+32	0.55	3.08
Citalopram	20	30.4	28.9	-5	25.8	-15	23.0	18.7	28.2	-24	-38	-7	0.007	3.08
Fluoxetine^d^	20	167.1	163.2	-2	154.4	-8	146.1	107.4	198.8	-13	-36	+19	0.39	3.23/3.39^e^
Sertraline	50	9.0	9.8	+10	12.2	+36	15.1	12.3	18.5	+68	+37	+106	<0.001	3.27
Venlafaxine^d^	100	141.8	135.8	-4	122.9	-13	111.2	79.6	155.4	-22	-44	+10	0.16	3.61/3.80^f^
Paroxetine	20	33.5	29.6	-12	22.1	-34	16.5	11.5	23.6	-51	-66	-30	<0.001	3.04
Fluvoxamine	100	117.9	101.9	-14	72.5	-38	51.6	29.3	91.1	-56	-75	-23	0.004	3.14

The column “baseline” provides the model estimates for the serum antidepressant concentrations at day 0 (non-pregnant). The first, second and third trimester columns provide the model estimates for the concentrations in the middle of these trimesters (gestational weeks 6, 20 and 34), respectively. The columns “change” provide the change from baseline concentration, in percent. Conc = concentration. CI = 95% confidence interval limits.

^a^ Dose = defined daily dose [28].

^b^ Serum concentrations in mass units can be converted to molar units by multiplication with the conversion factor (CF). Nanomol/L = ng/mL x CF

^c^ p-value for the regression line in the statistical model.

^d^ For drugs with clinically significant pharmacologically active metabolites the total active moiety concentrations were used for calculations (i.e. fluoxetine plus norfluoxetine, and venlafaxine plus O-desmethylvenlafaxine).

^e^ for fluoxetine and norfluoxetine, respectively.

^f^ for venlafaxine and O-desmethylvenlafaxine, respectively.

As the concentration distributions were found to be heavily right-skewed, the log_e_ of the concentrations was employed as the outcome variable in the statistical model to achieve near normality. Since multiple measurements were available from the same patient a linear mixed model was used. The model assumes that each individual patient possesses a random intercept (i.e. an individual “offset”) in addition to being affected by the gestational week at the time of sampling. Baseline measurements were set to gestational week 0 in the model. Then, the effect of gestational week on concentration compared to baseline could be estimated for each drug.

For drugs where both the parent drug and the metabolite were measured, parent drug/metabolite concentration ratios during pregnancy were compared to baseline values as described above; ratios were log_e_-transformed and fitted into a linear mixed model, estimating the baseline ratios and the effect of each gestational week.

All model parameters, including variance components, were estimated by the method of maximum likelihood using STATA 13 command “mixed”. Data are presented as means with 95% confidence intervals. P values less than 0.05 were considered statistically significant.

## Results

Table 1 and Fig 1 provide an overview of all analyses and pregnancies included in the study. The model estimates for the log_e_-transformed serum concentrations across pregnancy are given in the S1 Table. Table 2 shows the estimated serum concentrations at baseline and by trimester during pregnancy, as well as the relative changes from baseline in percent. For paroxetine, fluvoxamine and citalopram concentrations in mid third trimester (gestational week 34) were 51%, 56% and 24% lower than baseline values, respectively. For venlafaxine, fluoxetine and escitalopram the concentration declines were smaller and not statistically significant. For sertraline, there was a 68% increase in mid third trimester concentrations compared to baseline (Table 2).

**Fig 2:**
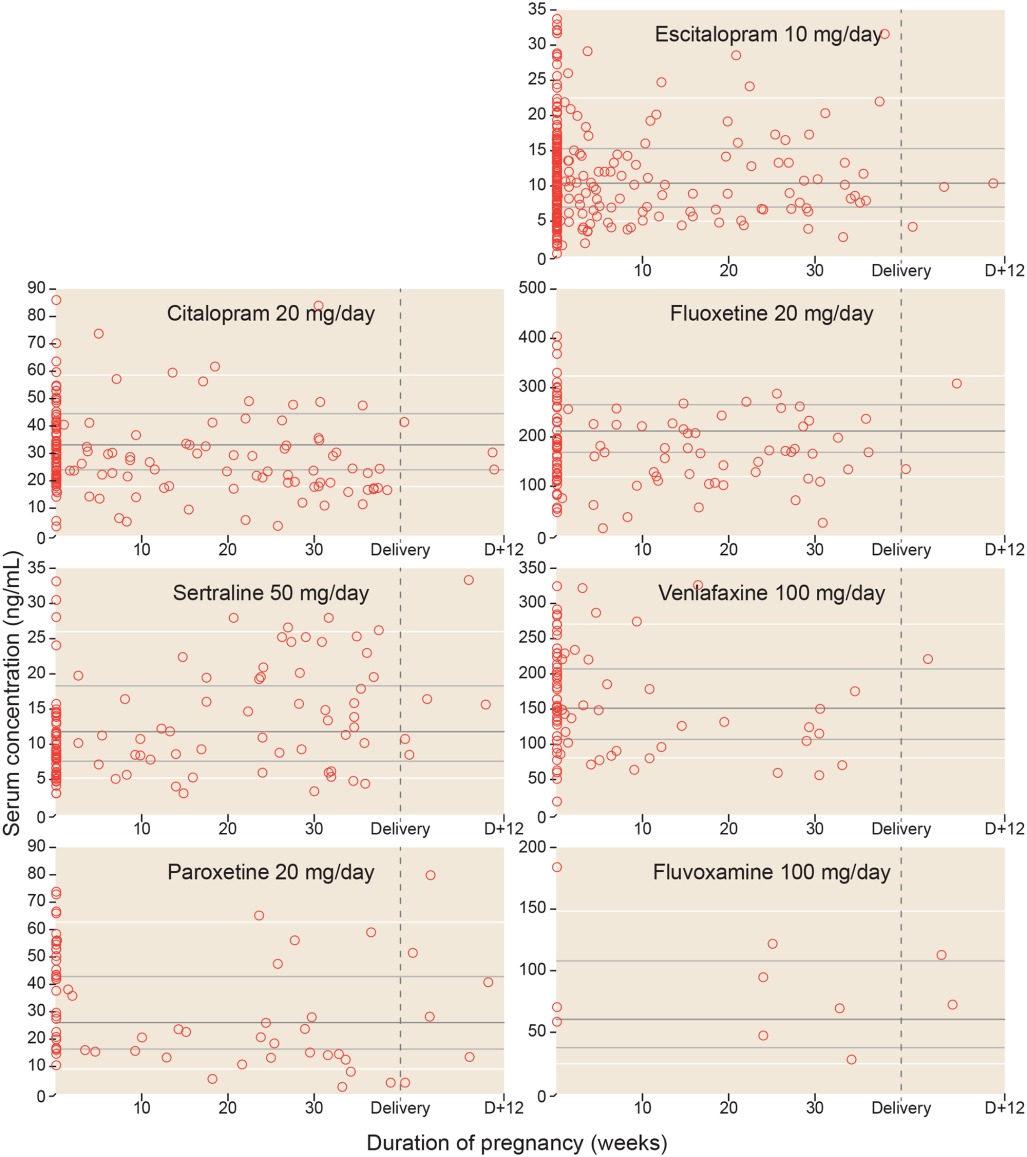
The serum antidepressant concentrations across pregnancy. The figure shows each of the observed serum concentrations in the study, adjusted to the doses presented in Table 2. Observations from the same women in non-pregnant state (baseline values) are shown as pregnancy week 0. Delivery is set to pregnancy week 40. Thus, for a woman who gave birth in week 38, a sample drawn x weeks after delivery would be shown x weeks to the right of the vertical delivery line. For fluoxetine and venlafaxine the concentrations shown represent the active moiety (parent drug + metabolite). Three outliers for escitalopram are not shown in the figure. These are one analysis in week 0 (concentration 36 ng/mL), one analysis in week 4 (concentration 36 ng/mL) and one analysis in week 5 (concentration 40 ng/mL). However, these concentrations are included in the statistical analyses. The horizontal lines represent the median (dark grey), 25 and 75 percentiles (light grey) and 10 and 90 percentiles (white) for dose-adjusted serum concentration measurements for all women aged 18–45 years from the St. Olav University Hospital TDM database. For further details, see Methods section.

**Fig 3:**
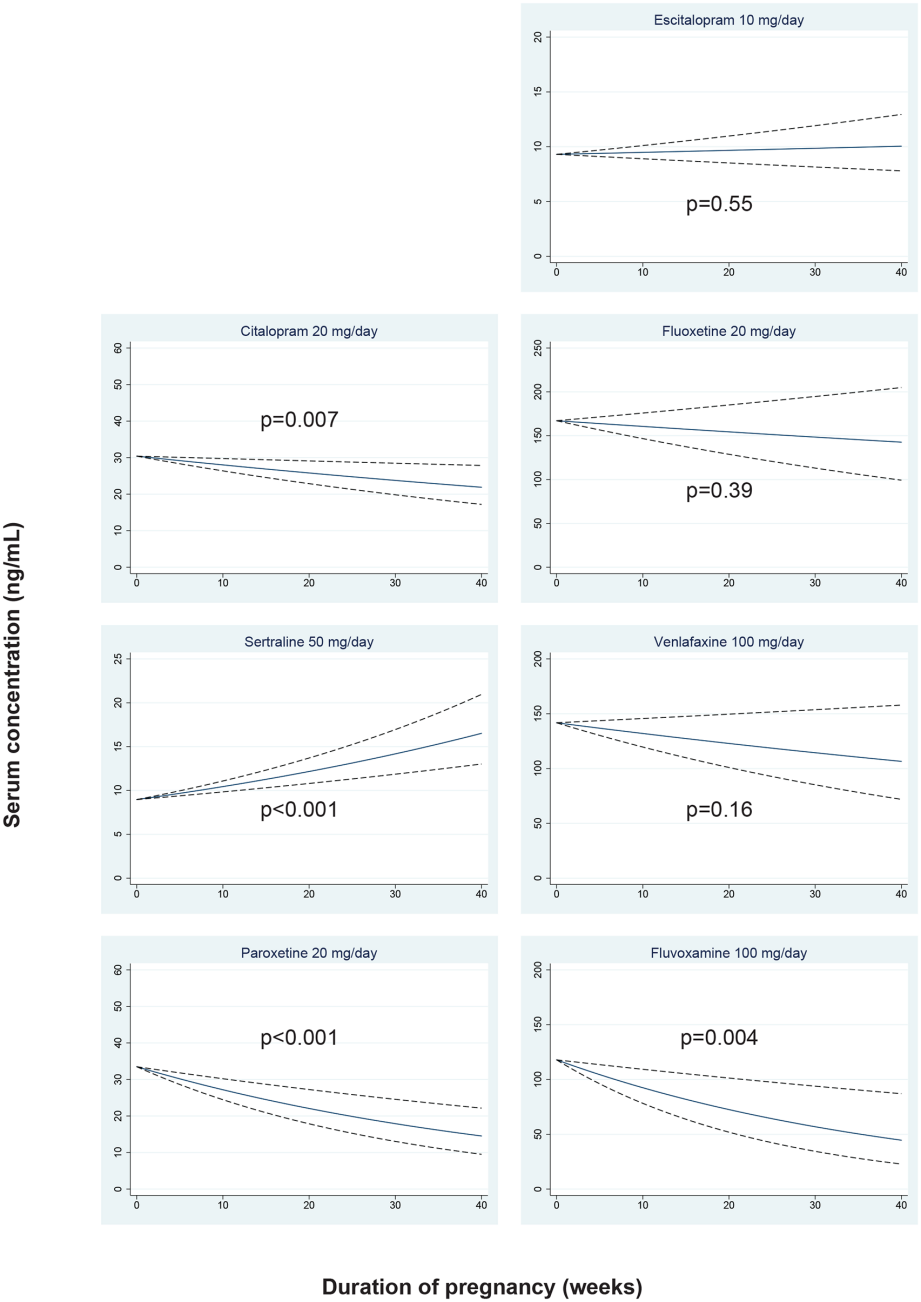
Regression lines for serum antidepressant concentrations across pregnancy. The figure shows the expected serum concentrations across pregnancy for women using the antidepressant doses presented in Table 2. The regression lines are shown in blue, and the 95% confidence limits with dashed black lines. For fluoxetine and venlafaxine the concentrations shown represent the active moiety (i.e. parent drug plus metabolite).

Individual concentrations related to gestational week, as well as when the women were not pregnant, are shown in Fig 2, together with the percentile values derived from the concentrations in the general female reference population. The measured concentrations in the time period from delivery to 12 weeks after delivery (i.e. in the “returning to baseline” phase) are also shown in Fig 2. The regression lines with 95% confidence limits showing the expected serum concentrations for each antidepressant drug during pregnancy are shown in Fig 3.

For escitalopram, citalopram, fluoxetine, sertraline and venlafaxine, metabolites had been measured in all or some samples, allowing us to study the parent compound / metabolite ratios. The original log_e_-transformed values (S2 Table) are converted to actual ratios in Table 3. For escitalopram, the parent compound / metabolite ratio in mid third trimester was 40% higher than baseline, whereas for fluoxetine and sertraline the mid third trimester ratios were 36% and 20% lower than baseline, respectively. There was also a trend towards a similar decline in parent compound / metabolite ratio for venlafaxine and citalopram, although the difference did not reach statistical significance (Table 3).

**Table 3:** Serum metabolite concentrations and parent compound/metabolite ratios at baseline and in the third trimester.

	Number of serum drug concentration analyses		Number of pregnancies (number of women)	Dose^a^ (mg/day)	Baseline conc.	Third trimester conc. (ng/mL)			Change from baseline conc.(%)			p^b^
	During pregnancy	At baseline			Estimate	Estimate	CI low	CI high	Estimate	CI low	CI high	
Escitalopram	63	98	61 (59)	10	8.4	10.5	7.5	14.6	+24	-11	+73	0.20
Desmethylescitalopram					4.7	4.1	3.2	5.2	-14	-32	+9	0.21
PMR					1.8	2.5	1.9	3.3	+40	+8	+82	0.012
Citalopram	50	26	37 (37)	20	32.3	19.7	14.5	26.7	-39	-55	-17	0.001
Desmethylcitalopram					12.6	8.5	6.6	11.0	-33	-48	-13	0.002
PMR					2.6	2.3	1.9	2.8	-13	-28	+5	0.16
Fluoxetine	53	49	43 (41)	20	77.7	53.3	34.5	82.3	-31	-56	+6	0.089
Norfluoxetine					83.7	83.7	61.6	112.8	0	-26	+35	0.98
PMR					1.0	0.6	0.4	0.9	-36	-55	-10	0.01
Sertraline	37	40	24 (21)	50	8.6	15.6	12.2	20.0	+83	+43	+133	<0.001
Desmethylsertraline					18.4	40.6	32.7	50.4	+120	+78	+173	<0.001
PMR					0.5	0.4	0.3	0.4	-20	-32	-5	0.009
Venlafaxine	36	44	33 (33)	100	35.8	21.4	12.6	36.1	-40	-65	+1	0.054
O-desmethylvenlafaxine					91.5	79.4	54.7	115.1	-13	-40	+26	0.45
PMR					0.4	0.3	0.2	0.5	-31	-59	+16	0.16

Only analyses with available metabolite data are included. The column “baseline conc.” provides the model estimates for the serum concentration of each parent compound, its metabolite, and the parent compound / metabolite ratio (PMR) at day 0 (non-pregnant), with 95% confidence interval limits. The “third trimester conc.” columns provide the model estimates for the same parameters in gestational week 34. The “change from baseline conc.” columns provide the change from baseline concentrations to third trimester concentrations, in percent. Conc = concentration. CI = 95% confidence interval limits.

^a^ Dose = defined daily dose.

^b^ p-value for the regression line in the statistical model.

## Discussion

The present study, including SSRI and venlafaxine serum concentration data from 290 pregnancies, is by far the largest study to date investigating the disposition of antidepressants during pregnancy. The main finding is that the serum concentrations of paroxetine and fluvoxamine drop to about 50% of pre-pregnancy levels, whereas sertraline concentrations increase by approximately 60–70% (Table 2). Venlafaxine, fluoxetine, citalopram and escitalopram concentrations remain largely unchanged.

Although a myriad of physiological changes that may alter drug disposition occur during pregnancy [4–7], total clearance is the primary determinant of the serum concentration at steady state. Since all drugs of our study are primarily eliminated by various hepatic cytochrome P450 (CYP) enzymes [32], we consider the activity of these enzymes to be the crucial explanatory factor for changes (or lack thereof) in the observed drug concentrations in our study.

Escitalopram disposition in pregnancy has previously been explored in five pregnancies in a study by Sit et al. [19]. They found only minor declines or no change in escitalopram concentrations throughout pregnancy. Our observations from 97 escitalopram pregnancies support those of Sit et al.; the concentration change estimate in our study was close to zero, with narrow confidence intervals. The clinical implication of our escitalopram findings is that dose adjustments is not expected to be necessary in pregnancy.

Citalopram is a chiral compound, consisting of S-citalopram (escitalopram, as described above) and the pharmacologically inactive R-citalopram [19]. The disposition of citalopram in pregnancy has previously been explored in two studies; Heikkinen et al. [13] found third trimester citalopram concentrations to be 42% lower than baseline values in 11 pregnancies, whereas Sit et al. [19] found third trimester concentrations to be 26% lower than baseline in two pregnancies. We found similar results; in our 58 citalopram pregnancies, there was a 24% reduction in third trimester concentrations compared to baseline. Interestingly, citalopram undergoes stereoselective metabolism; the pharmacologically active S-enantiomer (escitalopram) is metabolized primarily by CYP2C19 [33, 34], whose activity may *decrease* in pregnancy [6, 7], whereas the inactive R-enantiomer is metabolized primarily by CYP2D6 [35], whose activity *increases* in pregnancy [4–6]. Thus, it seems likely that the decline in citalopram concentrations during pregnancy was caused primarily by a decline in the inactive R-citalopram concentrations. On the basis of these findings, we recommend that citalopram doses—as for escitalopram—as a rule of thumb should be kept stable throughout pregnancy, even though the serum concentrations may decline throughout pregnancy.

Paroxetine is metabolized mainly by CYP2D6 and CYP3A4 [36]. Its disposition in pregnancy has previously been explored in two studies [17, 20]; Brogtrop et al. included 12 pregnancies and found lower concentrations in the third trimester compared to postpartum, although no numbers were provided [17]. Ververs et al. included 74 pregnancies and estimated the effect of gestational week on paroxetine plasma concentrations, in a statistical model similar to ours. Interestingly, by including genotype data, they found that changes in paroxetine disposition in pregnancy depended not only on gestational week, but also on CYP2D6 genotype. For ultrarapid or extensive CYP2D6 metabolizers maternal paroxetine plasma concentrations declined by 0.3 ng/mL per gestational week (which translates to a 30% reduction in week 34 for our data). For intermediate and poor CYP2D6 metabolizers concentrations *increased* by 0.6 ng/mL per gestational week [20], suggesting that other mechanisms dominate when CYP2D6 activity is low. In our study, with data from 20 pregnancies, there was a 51% reduction in third trimester concentrations compared to baseline. Genotyping was not available in our material, but we assume that our population consisted mainly of extensive CYP2D6 metabolisers, which is the most prevalent genotype in a Caucasian population [37]. Thus, as a general recommendation for paroxetine use during pregnancy, physicians should be aware that concentrations are most likely to decline throughout pregnancy, and that increased dose requirement (roughly 100% in the third trimester) might ensue for most, but not all patients. Close clinical monitoring in pregnancy is thus warranted, preferentially supported by serum concentration measurements and possibly also CYP2D6 genotyping if available.

Fluvoxamine pharmacokinetics in pregnancy has not been investigated previously. Fluvoxamine is predominantly metabolized by CYP2D6 and CYP1A2 [38]. Since CYP2D6 activity increases in pregnancy [4–6] while CYP1A2 activity decreases [6, 39], it has been hypothesized that these effects might counterbalance each other with regards to net fluvoxamine concentrations in pregnancy [38]. However, the results from our three pregnancies do not indicate that this is the case. We found concentrations in third trimester to be 56% lower than baseline, suggesting CYP2D6 induction to be the dominating effect in pregnancy. Thus, the clinical advice regarding follow-up and testing for pregnant women would be the same as for paroxetine above.

For fluoxetine, both the parent compound and its primary active metabolite norfluoxetine are chiral compounds [22]. The enzymatic conversion of fluoxetine to norfluoxetine is stereoselective; the S-enantiomer is demethylated mainly by CYP2D6, and the equipotent R-enantiomer mainly by CYP2C9 [40]. Heikkinen et al. reported plasma concentration measurements from 11 pregnancies and found that third trimester concentrations of the active moiety (fluoxetine plus norfluoxetine) were 32% lower than baseline. They also found that the decline affected mainly the parent drug and to a lesser degree the metabolite. Similar observations were made in a study of nine pregnancies by Kim et al. [15], and 17 pregnancies by Sit et al. [22], who both also performed chiral analysis and found that S-fluoxetine concentrations declined more than R-fluoxetine in pregnancy. In our study chiral analyses were not undertaken, but our large sample size (43 pregnancies) supports the findings from previous studies in that fluoxetine concentrations decline in pregnancy, whereas norfluoxetine concentrations remain largely unchanged (Table 3). For the sum of the active moiety no major decline was observed in our study (Table 2). The stereoselective fluoxetine disposition in pregnancy reported from previous studies [15, 22] (i.e. increased CYP2D6-induced bioconversion from S-fluoxetine to S-norfluoxetine, who are both pharmacologically active) may explain why antidepressant response did not deteriorate during pregnancy in previous studies [14, 22]. We therefore suggest, as a rule of thumb, that fluoxetine doses could be kept stable throughout pregnancy.

Venlafaxine is metabolized by CYP2D6 to its equipotent metabolite O-desmethylvenlafaxine (ODVM) [24]. In a case report by Klier et al., a more than 50% reduction in venlafaxine plasma levels was observed in pregnancy compared to baseline [16]. However, in a prospective study of seven pregnancies by ter Horst et al., only a 13% reduction in venlafaxine levels in pregnancy was found, with no change in ODMV levels [24]. Our study, with 33 pregnancies, confirms the latter observation; we found a trend towards a statistically significant decline in venlafaxine concentrations, but the metabolite concentrations did not change (Table 3), and the changes in total active moiety levels were not statistically significant (Table 2). These results may reflect increased CYP2D6-induced bioconversion from venlafaxine to ODMV in pregnancy. This shift is expected to be of minor or no clinical relevance, since parent drug and metabolite share equal antidepressant potency [24]. We therefore suggest venlafaxine doses could be kept stable throughout pregnancy.

For sertraline, in contrast to the other antidepressants, we found a statistically significant *increase* in serum concentrations in pregnancy compared to baseline (Table 2). Sertraline is metabolized by multiple enzymes, including CYP2B6, CYP2C19, CYP2C9, CYP2D6, monoamine oxidases, and several UGT enzymes [34, 41]. The effect of pregnancy on these enzymes is divergent and to some extent unknown [6]. However, since we found increasing levels of both sertraline and the metabolite desmethylsertraline in pregnancy (Table 3), we suspect CYP2C19 inhibition [41] to play a crucial role. Previous studies on sertraline disposition in pregnancy have been limited by small sample sizes (eight and six pregnancies, respectively [18, 19]) and variable/non-significant observations; some women had decreasing sertraline concentrations in pregnancy, some remained stable, and a few had increasing concentrations [18, 19]. The authors of one of these studies [18] suggested that genetic factors might explain the observed heterogeneity. However, in our study the changes did not appear very heterogeneous. The increasing concentrations were a general trend in the population and were not caused by outlier observations (S1 Fig) or by differences in sampling time (S3 Table). Still, due to the relatively wide reference range and low toxicity of sertraline [34], increasing concentrations do not necesarily imply a need for dose reduction. We therefore recommend that patients as a rule of thumb remain on their usual sertraline dose in pregnancy, and that dose adjustments should be made on the basis of clinical follow-up, if available combined with therapeutic drug monitoring.

For all therapeutic drugs used in pregnancy, it is also important to explore when and how maternal serum concentrations return back to normal following delivery. Some researchers have provided evidence of a postpartum drop in metabolic capacity that could result in briefly elevated concentrations (i.e. higher than baseline) of some antidepressants during the first 6–8 weeks following delivery [10, 13, 19, 22, 42]. Due to relatively few postpartum observations our study can neither conclusively confirm nor rule out that such a refractory period occurs, although our results indicate that serum concentrations return back to baseline values within the first weeks after delivery (Fig 2).

Our study has some limitations that need to be addressed. First, as we did not have access to any clinical data we do not know whether the reduced concentrations for some of the antidepressants actually caused clinical deterioration. Although it is reasonable to assume that this could occur, and some studies have provided evidence for a correlation between declining antidepressant concentrations and clinical deterioration in pregnancy [8, 9, 16, 19, 20, 22], others have failed to detect such a relationship [13, 14, 17, 25]. Thus, we need future studies to address and explore the clinical consequences of the changing pharmacokinetics of antidepressants in pregnancy.

Second, it is unknown to which degree patients were adherent to the prescribed medication; a particular challenge during pregnancy [43]. However, all analyses with a serum concentration of zero (n = 19, Fig 1) were excluded from the study, and even though an increased degree of non-adherence during pregnancy would cause lower concentrations, we consider it being unlikely that such a situation should be confined to paroxetine, fluvoxamine and citalopram, and not for instance sertraline or escitalopram.

Third, the reason for why each serum concentration measurement was undertaken was in most cases unknown. Thus, due to the naturalistic nature of the study there is a possibility for selection bias in observations, e.g. an overrepresentation of pregnancy samples taken from patients with treatment failure. However, our impression is that serum concentration measurements in pregnancy are conducted in the same way as in non-pregnant patients, and that most samples are taken as routine follow-up. Also, we consider it being unlikely that such a selection bias should be confined to some drugs only.

Forth, there is a variability of the time interval from last dose to sampling. Ideally, this interval should have been standardized to 12 hours, and all values calculated to such using drug-specific elimination half-lives, as in a previous publication from our group [44]. However, the information needed for calculating the time interval was often missing on the requisition form, and excluding all such analyses would result in loss of precious data. We believe that some of the variability in our results (Fig 2) derives from variations in these time intervals, an inevitable factor given the retrospective nature of our study, but we found no systematical differences in the post-dose time interval for serum concentration measurements between pregnant and non-pregnant women (S3 Table).

On the other hand, this study also has some strengths, the most obvious being the very large sample size. Due to the ethical issues involved in clinical drug trials during pregnancy [45, 46], retrospective studies of samples taken in a naturalistic setting is one the very few available tools to obtain information on drug disposition in pregnancy. Due to the variability often seen in observational studies a large sample size is crucial, such as our use of data from two large routine TDM services over a time span of 12 years. It is also a strength that we could link the TDM data a national birth registry, thereby allowing precise identification of pregnant women in the data set, and making misclassification of gestational week highly unlikely.

In conclusion, our results show that in order to maintain stable serum drug concentrations in pregnancy, paroxetine and fluvoxamine doses may need to be roughly doubled in the third trimester. For escitalopram, citalopram, fluoxetine, venlafaxine and sertraline, dose adjustments are generally not necessary in pregnancy. If available, therapeutic drug monitoring could be a useful supplement to the individual clinical evaluation in pregnancy, in order to determine optimum dose for each patient. This applies to all drugs of the study, although most important for the drugs that seem to undergo the greatest changes (paroxetine, fluvoxamine and sertraline). Therapeutic drug monitoring should preferentially begin when the woman is well prior to or in an early stage of pregnancy. The measured drug level could be used as that woman’s target concentration across pregnancy, in a similar approach as is already used for lamotrigine and other anticonvulsants [47].

## Supporting information

S1 Table The model parameter estimates for log_e_ serum antidepressant concentrations. The “Intercept” columns show the model estimates for log_e_ serum concentrations (dose-adjusted) at day 0 in the column “Estimate”, and the corresponding confidence limits and p-values for each drug estimated. The “Change per gestational week” columns provide an estimate for the change in the log_e_ serum concentration for each gestational week, with corresponding confidence limits and p-values for each drug. The estimated concentration in gestational week *t* is thus calculated by the following equation: Serum concentration (week *t*) = e^the intercept estimate + (*t* ∙ change per gestational week estimate)^. Table 2 provides an overview of the estimated concentrations for each trimester.
CI = confidence interval
^a^ For drugs with clinically significant pharmacologically active metabolites the total active moiety concentrations were used for calculations (i.e fluoxetine plus norfluoxetine, and venlafaxine plus O-desmethylvenlafaxine).
(DOCX)

S2 Table The model parameter estimates for log_e_ serum concentrations of parent compounds and their metabolites. Only analyses with available metabolite data (see Table 3) are included. The model estimates for the log_e_ serum concentrations (adjusted to the doses presented in Table 3) for each antidepressant and their metabolites, and the log_e_-transformed ratio between parent compound and metabolite (PMR). The “intercept” columns provide the baseline estimate (i.e. day 0 of pregnancy), and the corresponding confidence interval (CI) limits and p-values for each drug estimated. The “Change per gestational week” columns provide an estimate for the change in log_e_ concentration or log_e_ PMR for each week of pregnancy, with corresponding confidence limits and p-values for each drug estimated. The estimated serum concentration (or PMR) in gestational week *t* is thus calculated by the following equation: Serum concentration (week *t*) = e^the intercept estimate + (*t* ∙change per gestational week estimate)^. Table 3 provides an overview of calculated serum concentrations and PMR for each trimester.
(DOCX)

S3 Table Mean post-dose time intervals for serum concentration measurements. (DOCX)

S1 Fig Individual sertraline concentrations in pregnancy (n = 56). The figure displays the same sertraline serum concentrations as in Fig 2, but with separate symbols/colours for each subject.
(DOCX)
