# Correction: Selective serotonin reuptake inhibitors and venlafaxine in pregnancy: Changes in drug disposition

**DOI:** 10.1371/journal.pone.0191508

**Published:** 2018-01-16

**Authors:** Andreas Austgulen Westin, Malin Brekke, Espen Molden, Eirik Skogvoll, Olav Spigset

The affiliation for the 1st author is incomplete. Andreas Austgulen Westin is affiliated with #1 and #6.

There is an error in the caption for Fig 1, “Inclusion flow chart.” Please see the complete, correct Fig 1 caption here.

**Fig 1:**
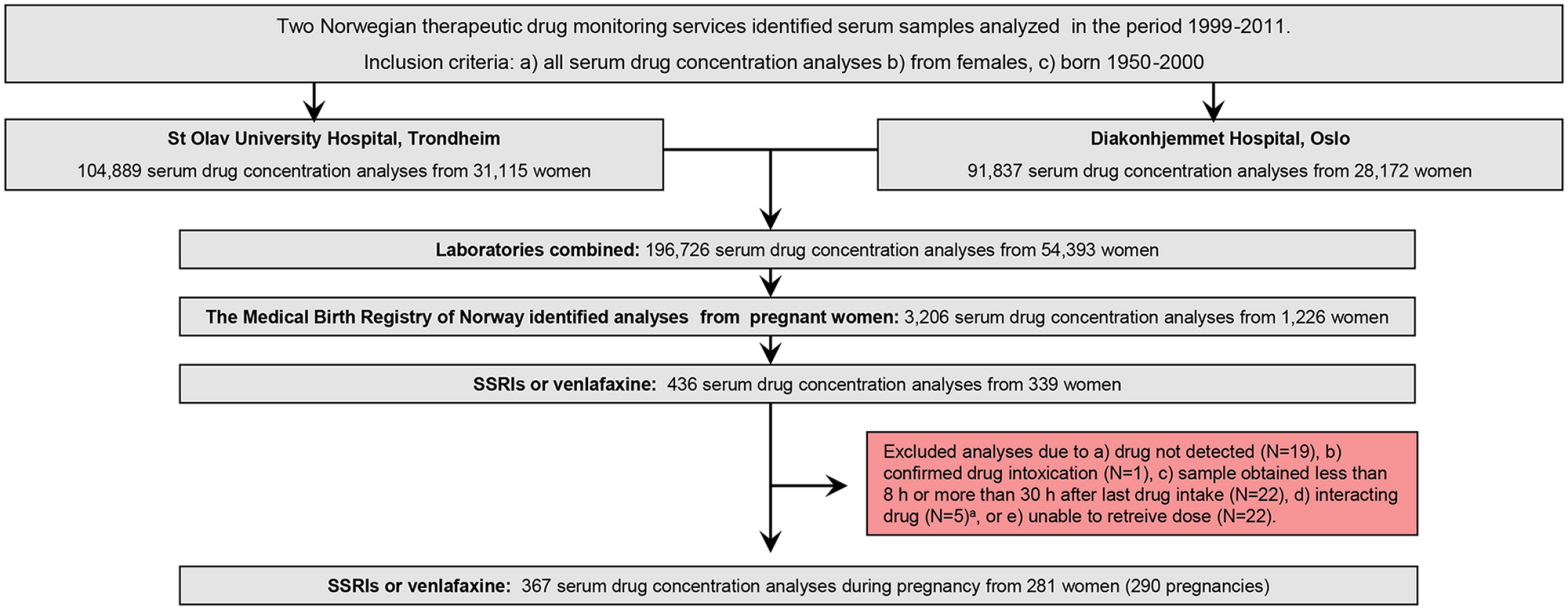
Inclusion flow chart. Sample identification and inclusion of therapeutic drug monitoring samples of selective serotonin reuptake inhibitors and venlafaxine obtained during pregnancy. ^a^ Five analyses were excluded due to the following drug interactions: sertraline + carbamazepine (n = 1), venlafaxine + bupropion (n = 4).
