# Supplementary material for: Selective serotonin reuptake inhibitors and venlafaxine in pregnancy: Changes in drug disposition
Source: PLoS One. 2017 Jul 14;12(7):e0181082. doi: 10.1371/journal.pone.0181082 (PMC5510868; doi:10.1371/journal.pone.0181082)
Supplement: S1 Table — The “Intercept” columns show the model estimates for loge serum concentrations (dose-adjusted) at day 0 in the column “Estimate”, and the corresponding confidence limits and p-values for each drug estimated. The “Change per gestational week” columns provide an estimate for the change in the loge serum concentration for each gestational week, with corresponding confidence limits and p-values for each drug. The estimated concentration in gestational week t is thus calculated by the following equation: Serum concentration (week t) = ethe intercept estimate + (t ∙ change per gestational week estimate). Table 2 provides an overview of the estimated concentrations for each trimester. CI = confidence interval a For drugs with clinically significant pharmacologically active metabolites the total active moiety concentrations were used for calculations (i.e fluoxetine plus norfluoxetine, and venlafaxine plus O-desmethylvenlafaxine). (DOCX) [file pone.0181082.s001.docx]

**S1 Table. The model parameter estimates for log_e_ serum antidepressant concentrations**

|  |  | Log_e_ serum drug concentration (in ng/mL) | | | | | | | |
| --- | --- | --- | --- | --- | --- | --- | --- | --- | --- |
|  | Dose | Intercept | | | | Change per gestational week | | | |
| Measure | mg/day | Estimate | 95% CI low | 95% CI high | p | Estimate | 95% CI low | 95% CI high | p |
| Escitalopram | 10 | 2.230 | 2.101 | 2.360 | <0.001 | 0.002 | -0.004 | 0.008 | 0.55 |
| Citalopram | 20 | 3.415 | 3.270 | 3.559 | <0.001 | -0.008 | -0.014 | -0.002 | 0.007 |
| Fluoxetine^a^ | 20 | 5.119 | 4.960 | 5.277 | <0.001 | -0.004 | -0.013 | 0.005 | 0.39 |
| Sertraline | 50 | 2.193 | 1.990 | 2.396 | <0.001 | 0.015 | 0.009 | 0.021 | <0.001 |
| Venlafaxine^a^ | 100 | 4.954 | 4.777 | 5.131 | <0.001 | -0.007 | -0.017 | 0.003 | 0.16 |
| Paroxetine | 20 | 3.512 | 3.245 | 3.778 | <0.001 | -0.021 | -0.031 | -0.010 | <0.001 |
| Fluvoxamine | 100 | 4.770 | 4.179 | 5.361 | <0.001 | -0.024 | -0.041 | -0.008 | 0.004 |

The “Intercept” columns show the model estimates for log_e_ serum concentrations (dose-adjusted) at day 0 in the column “Estimate”, and the corresponding confidence limits and p-values for each drug estimated. The “Change per gestational week” columns provide an estimate for the change in the log_e_ serum concentration for each gestational week, with corresponding confidence limits and p-values for each drug. The estimated concentration in gestational week *t* is thus calculated by the following equation: Serum concentration (week *t*) = e^the intercept estimate + (^*^t^* ^∙ change per gestational week estimate)^. Table 2 provides an overview of the estimated concentrations for each trimester.

CI = confidence interval

^a^ For drugs with clinically significant pharmacologically active metabolites the total active moiety concentrations were used for calculations (i.e fluoxetine plus norfluoxetine, and venlafaxine plus O-desmethylvenlafaxine).
