# Supplementary material for: Selective serotonin reuptake inhibitors and venlafaxine in pregnancy: Changes in drug disposition
Source: PLoS One. 2017 Jul 14;12(7):e0181082. doi: 10.1371/journal.pone.0181082 (PMC5510868; doi:10.1371/journal.pone.0181082)
Supplement: S2 Table — Only analyses with available metabolite data (see Table 3) are included. The model estimates for the loge serum concentrations (adjusted to the doses presented in Table 3) for each antidepressant and their metabolites, and the loge-transformed ratio between parent compound and metabolite (PMR). The “intercept” columns provide the baseline estimate (i.e. day 0 of pregnancy), and the corresponding confidence interval (CI) limits and p-values for each drug estimated. The “Change per gestational week” columns provide an estimate for the change in loge concentration or loge PMR for each week of pregnancy, with corresponding confidence limits and p-values for each drug estimated. The estimated serum concentration (or PMR) in gestational week t is thus calculated by the following equation: Serum concentration (week t) = ethe intercept estimate + (t ∙change per gestational week estimate). Table 3 provides an overview of calculated serum concentrations and PMR for each trimester. (DOCX) [file pone.0181082.s002.docx]

**S2 Table. The model parameter estimates for log_e_ serum concentrations of parent compounds and their metabolites**

|  | Log_e_ serum concentration, or log_e_ PMR | | | | | | | |
| --- | --- | --- | --- | --- | --- | --- | --- | --- |
|  | Intercept | | | | Change per gestational week | | | |
|  | estimate | 95%  CI low | 95%  CI high | p | estimate | 95%  CI low | 95%  CI high | p |
| Escitalopram  Desmethylescitalopram  PMR | 2.129  1.557  0.581 | 1.964  1.405  0.417 | 2.295  1.710  0.745 | <0.001  <0.001  <0.001 | 0.006  -0.004  0.010 | -0.003  -0.011  0.002 | 0.016  0.002  0.018 | 0.20  0.21  0.012 |
| Citalopram  Desmethylcitalopram  PMR | 3.475  2.534  0.973 | 3.275  2.363  0.826 | 3.676  2.706  1.119 | <0.001  <0.001  <0.001 | -0.015  -0.012  -0.004 | -0.024  -0.019  -0.010 | -0.006  -0.004  0.002 | 0.001  0.002  0.16 |
| Fluoxetine  Norfluoxetine  PMR | 4.353  4.427  -0.045 | 4.141  4.237  -0.262 | 4.565  4.617  0.173 | <0.001  <0.001  <0.001 | -0.011  0.000  -0.013 | -0.024  -0.009  -0.023 | 0.002  0.009  -0.003 | 0.089  0.98  0.01 |
| Sertraline  Desmethylsertraline  PMR | 2.147  2.914  -0.764 | 1.893  2.720  -0.896 | 2.400  3.108  -0.631 | <0.001  <0.001  <0.001 | 0.018  0.023  -0.006 | 0.010  0.017  -0.011 | 0.025  0.030  -0.002 | <0.001  <0.001  0.009 |
| Venlafaxine  O-desmethylvenlafaxine  PMR | 3.577  4.516  -0.927 | 3.239  4.336  -1.287 | 3.914  4.697  -0.566 | <0.001  <0.001  <0.001 | -0.015  -0.004  -0.011 | -0.031  -0.015  -0.026 | 0.000  0.007  0.004 | 0.054  0.45  0.16 |

Only analyses with available metabolite data (see Table 3) are included. The model estimates for the log_e_ serum concentrations (adjusted to the doses presented in Table 3) for each antidepressant and their metabolites, and the log_e_-transformed ratio between parent compound and metabolite (PMR). The “intercept” columns provide the baseline estimate (i.e. day 0 of pregnancy), and the corresponding confidence interval (CI) limits and p-values for each drug estimated. The “Change per gestational week” columns provide an estimate for the change in log_e_ concentration or log_e_ PMR for each week of pregnancy, with corresponding confidence limits and p-values for each drug estimated. The estimated serum concentration (or PMR) in gestational week *t* is thus calculated by the following equation: Serum concentration (week *t*) =e^the intercept estimate + (^*^t^* ^∙change per gestational week estimate)^. Table 3 provides an overview of calculated serum concentrations and PMR for each trimester.
