# Supplementary material for: Selective serotonin reuptake inhibitors and venlafaxine in pregnancy: Changes in drug disposition
Source: PLoS One. 2017 Jul 14;12(7):e0181082. doi: 10.1371/journal.pone.0181082 (PMC5510868; doi:10.1371/journal.pone.0181082)
Supplement: S3 Table — (DOCX) [file pone.0181082.s003.docx]

**S3 Table. Mean post-dose time intervals for serum concentration measurements**

|  | Phase | Post dose time (hours, mean ± SD) |
| --- | --- | --- |
| Escitalopram | Pregnancy  Baseline | 20.1 ± 5.7  20.9 ± 6.0 |
| Citalopram | Pregnancy  Baseline | 19.7 ± 5.7  20.1 ± 5.9 |
| Fluoxetine | Pregnancy  Baseline | 20.2 ± 6.0  19.5 ± 6.6 |
| Sertraline | Pregnancy  Baseline | 18.3 ± 6.4  19.6 ± 5.4 |
| Venlafaxine | Pregnancy  Baseline | 19.1 ± 5.9  18.4 ± 6.3 |
| Paroxetine | Pregnancy  Baseline | 20.9 ± 5.4  22.4 ± 5.2 |
| Fluvoxamine | Pregnancy  Baseline | 13.0 ± 0.8  12.4 ± 0.4 |
