# Supplementary material for: Selective serotonin reuptake inhibitors and venlafaxine in pregnancy: Changes in drug disposition
Source: PLoS One. 2017 Jul 14;12(7):e0181082. doi: 10.1371/journal.pone.0181082 (PMC5510868; doi:10.1371/journal.pone.0181082)
Supplement: S1 Fig — The figure displays the same sertraline serum concentrations as in Fig 2, but with separate symbols/colours for each subject. (DOCX) [file pone.0181082.s004.docx]

**S1 Fig. Individual sertraline concentrations in pregnancy (n = 56)**

Serum concentration (ng/mL)

Duration of pregnancy (weeks)

The figure displays the same sertraline serum concentrations as in Figure 2, but with separate symbols/colours for each subject.
